# Multi-Zone Authentication and Privacy-Preserving Protocol (MAPP) Based on the Bilinear Pairing Cryptography for 5G-V2X

**DOI:** 10.3390/s21020665

**Published:** 2021-01-19

**Authors:** Shimaa A. Abdel Hakeem, HyungWon Kim

**Affiliations:** 1 School of Electronics Engineering, Chungbuk National University, Cheongju 28644, Korea; shimaakotb@cbnu.ac.kr; 2 Electronics Research Institute (ERI), Giza 12622, Egypt

**Keywords:** vehicular communication, security of bilinear pairing, privacy issues, authentication requirements, signatures aggregation, signature concatenation, cellular-V2X, multi-hop authentication

## Abstract

5G-Vehicle-to-Everything (5G-V2X) supports high-reliability and low latency autonomous services and applications. Proposing an efficient security solution that supports multi-zone broadcast authentication and satisfies the 5G requirement is a critical challenge. In The 3rd Generation Partnership Project (3GPP) Release 16 standard, for Cellular- Vehicle-to-Everything (C-V2X) single-cell communication is suggested to reuse the IEEE1609.2 security standard that utilizes the Public Key Infrastructure (PKI) cryptography. PKI-based solutions provide a high-security level, however, it suffers from high communication and computation overhead, due to the large size of the attached certificate and signature. In this study, we propose a light-weight Multi-Zone Authentication and Privacy-Preserving Protocol (MAPP) based on the bilinear pairing cryptography and short-size signature. MAPP protocol provides three different authentication methods that enable a secure broadcast authentication over multiple zones of large-scale base stations, using a single message and a single short signature. We also propose a centralized dynamic key generation method for multiple zones. We implemented and analyzed the proposed key generation and authentication methods using an authentication simulator and a bilinear pairing library. The proposed methods significantly reduce the signature generation time by 16 times–80 times, as compared to the previous methods. Additionally, the proposed methods significantly reduced the signature verification time by 10 times–16 times, as compared to the two previous methods. The three proposed authentication methods achieved substantial speed-up in the signature generation time and verification time, using a short bilinear pairing signature.

## 1. Introduction

Vehicle-to-Everything (V2X) communication [1] is the technology for connected vehicles to support road safety and prevent traffic accidents. V2X allows vehicles to broadcast periodic messages about the surrounding area. Recently, many technologies target road safety with high data rates to provide alerts about upcoming crashes. Multiple accessing technologies provide connectivity in vehicular networks, such as Wi-Fi, IEEE 802.11p, and cellular radio communications. Recently, Cellular-V2X (C-V2X) was standardized by the third-generation partnership project (3GPP) for automotive services. LTE-V2X is the current 3GPP Release 14 [2] standard that has many enhancements to provide the new 3GPP Release 16 for the new 5G radio generation [3]. The majority of the shortcomings of DSRC, 802.11p, and LTE-V2X are meant to be handled through the efficient function handlers in 5G-V2X. Proposing efficient light-weight security solutions against known and unknown threats depends on the deployment strategies of 5G-V2X. The deployment of 5G Base Stations (BSs) defines the exploitation of possible network vulnerabilities. Moreover, key exposure and the insecure communication channel were considered as points of attack in 5G-V2X. Due to the high mobility conditions, attack possibilities are increasing. Optimizing the 5G-NR in V2X communication has high requirements and efficient light-weight security methods to support many V2X services and applications [4]. The 5G NewRadio (NR) was developed to enhance the network scalability, flexibility, and efficiency of the spectrum and power usage [5]. V2X communication offers different benefits, but it creates many privacy and security concerns [6]. Many proposed security protocols are trying to satisfy the security standards requirements and challenges [7]. V2X authentication and privacy are the most critical issues that are our basic concerns in this study. A few studies looked at the security requirements of C-V2X networks [8,9]. C-V2X security standards in the 3GPP Release 15 and beyond are based on the Public Key Infrastructure (PKI), to preserve privacy and support message authentication. PKI-based solutions suffer high communication and computation overhead due to the large size of the attached certificate and signature [10]. 5G-V2X infrastructure plays a vital role in designing efficient security protocol. The intensive deployment of 5G BSs at short distances can serve as Road Side Units (RSUs) to offer security services for road vehicles [11]. Each vehicle authenticates itself to the joined cell BS before communication. The handover authentication between BSs consumes long delays to transfer the authentication parameters of vehicles between different cells. Moreover, in 3GPP Release 15, LTE-V2X supports 100 ms End-to-End latency that can allow vehicles to communicate with BSs, request the authentication parameters, and authenticate messages within this time. However, in Release 16, for the 5G-V2X it was assumed that latency was 5 ms or less, which required fast authentication procedures to satisfy the 5G requirements. One enhancement introduced by the 5GCAR project was related to the concept of zones as a solution to provide common local services using BSs as roadside units (RSUs). As considered in 5GCAR, the BSs were grouped to form smart radio access service areas, referred to as Smart Zones (SM-Zones) [12]. In this study, we assumed that the 5G-V2X network is divided into N zones, and every zone is covered by n BSs. For a single zone, all vehicles were configured with common security parameters to securely communicate without high-cost re-authentication, when they moved from one BS to another.

We propose a Multi-Zone Authentication and Privacy-Preserving Protocol (MAPP), based on the bilinear pairing cryptography and a short digital signature. The proposed protocol supports the message and identity authentication within single-zone and multiple zones that enhance the 5G-V2X network security and availability.

In summary, the contributions of this study are as follows:

- Proposing a dynamic key generation method that provides short-lived authentication keys per vehicle in each zone.
- Proposing a Transmitter Centric Authentication (TCA) method where signature generation at transmitters and signature verification at receivers are based on the transmitter zone parameters.
- Proposing a Signature Concatenation-based Authentication (SCA) method, in which the transmitter generates a concatenated signature that can be individually verified by all receivers, using their corresponding zone parameters.
- Proposing a Receiver Centric Authentication (RCA) method, where transmitters and receivers aggregate the security parameters of the overlapped zones to generate and verify signatures.
- Comparing the three proposed authentication methods in terms of signature generation time, signature verification time, and communication cost.
- Comparing the communication cost in terms of message size for the three proposed authentication methods and six previous related methods for single-zone and multi-zone scenarios.
- Comparing the computation cost in terms of signature generation time and signature verification.

The rest of this paper is organized as follows. Section 2 describes the previous V2X security methods based on the bilinear pairing and free of certificates solutions. Section 3 presents the proposed protocol architecture and the three proposed authentication methods. Section 4 describes security analysis, and the proposed communication overhead analysis is presented in Section 5. In Section 6, the computation overhead is analyzed. Conclusions are provided in Section 7.

## 2. Related Work

Many security methods are proposed to support authentication for all exchanged periodic information in V2X. Public Key Infrastructure (PKI) authentication methods were proposed to support message authentication using digital signatures and identity authentication using a certificate [13]. The digital signatures in PKI provide authentication and integrity, using long size certificates incurs high communication and computation overhead. Due to the high-cost of PKI-based methods, some identity-based (ID-based) authentication methods are proposed in [14,15,16,17].

In [14], He et al. proposed an identity-based privacy-preserving authentication method for V2X. In [15], Lo et al. designed a new ID-based authentication method using Elliptic Curve (ECC) for authentication and privacy-preserving. These ID-based methods require less communication overhead to support authentication and preserve privacy. In [16], Liu et al. presented an efficient anonymous authentication method using message recovery and signatures to enhance system efficiency. In [17], Tzeng et al. proposed the batch verification method based on identity authentication for V2X and defined different security risks. In [18], Hu et al. improved the proposed Tzeng et al. [17] methods, by proposing a secure batch verification method based on ID, without bilinear pairings. Though these ID-based solutions could eliminate the PKI problems, it suffers from a key escrow problem. To overcome the PKI overhead and the key escrow problems of ID-based solutions, many certificateless (CLS) signature methods were proposed [19,20,21,22,23,24]. Horng et al. [19] proposed a privacy-preserving aggregated signatures method for V2V communication. In this method, only the partial private key of the users was generated by a trusted Key Generator Center (KGC). A secret random value was picked by each user and combined with the partial private key to generate a new private key. Therefore, the user’s private keys were not stored at the KGC. In certificateless CPPA methods, vehicles do not need to store certificates to guarantee the authenticity of the used public keys. Li et al. [20] proved that the proposed method in [19] was not secure against the passive malicious KGC, using the existing security model. Malhi et al. [21] proposed a new efficient certificateless aggregate signature protocol for V2X, and proved the security level using the random oracle model. Additionally, the proposed protocol was computationally more efficient due to its constant pairing operations. After discussing the vulnerabilities of malicious-but-passive KGC attacks, Lin et al. [22] presented an improved protocol. They presented a new security method, based on authentication using group signatures for V2X. In this method, a single manager issues the secret keys for each vehicle. Bayat et al. [23] proposed a new Conditional Privacy-Preserving Authentication (CPPA) method, based on bilinear pairing cryptography, to improve identity-based authentication in V2X. However, this method could not prevent the message modification attacks in which an attacker could repeat the transmission of old messages after modifying its content. In [24], Boneh et al. proposed the first protocol for group signature, based on bilinear pairing. This group signature protocol suffers from high computation and communication cost. All mentioned methods in [19,20,21,22,23,24], employ the bilinear pairing cryptography for a single cell or group authentication. It also limits the network scalability and availability, and suffers from high computation time and complexity. We summarize the advantages and disadvantages of the security protocols [19,20,21,22,23,24] in Table 1.

In this study, we proposed a Multi-Zone Authentication and Privacy-Preserving Protocol (MAPP), based on bilinear pairing cryptography. The proposed protocol supports privacy-preserving by generating pseudo-identities to hide the vehicle’s real identities. MAPP provides broadcast authentication methods over multiple-zones, using short bilinear pairing signatures. We utilize special elliptical curves to reduce the authentication time and storage requirements. MAPP supports dynamic key generation per vehicle in each zone that enhances the security level and resists the key attacks in previous V2X methods.

## 3. The Proposed Protocol

**Figure 1:**
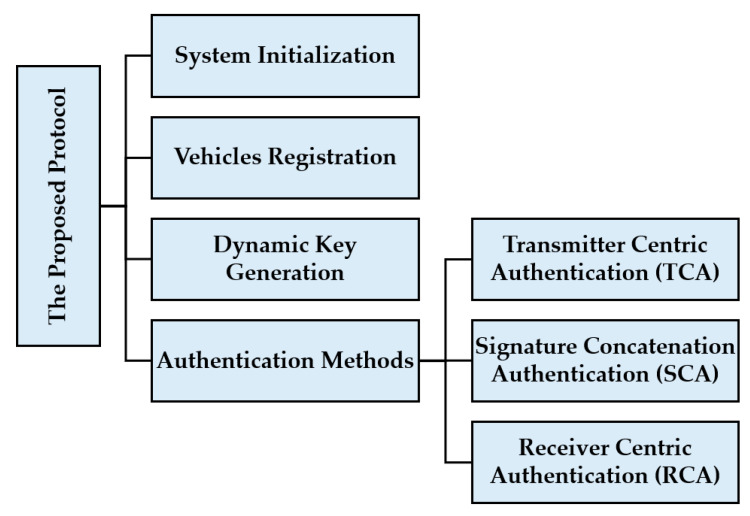
The proposed protocol architecture.

In this section, we describe the proposed protocol with the following steps—a system model, system initialization, and the proposed authentication methods. Figure 1 summarizes the proposed protocol architecture that introduces three new authentication methods. Table 2 summarizes the system notations and the commonly mentioned variables.

### 3.1. System Model

5G is the next mobile radio generation that supports ultra-high data speeds and low latency [25]. It was predicted that the number of 5G devices would be high, with high generated traffic [26]. Thus, there is a critical need for the improvement of cell deployment. The 5G new antenna techniques use the mmWave carrier frequencies. mmWave offers a short range of communication with a large amount of data [27]. There are several advantages of using mmWave frequencies in 5G networks, such as privacy and security, due to the short transmission range of mmWave, and also reusing the same frequency in a very short distance. Thus, many Base Stations should be placed at short distances to offer local management services with better frequency reuse [28].

**Figure 2:**
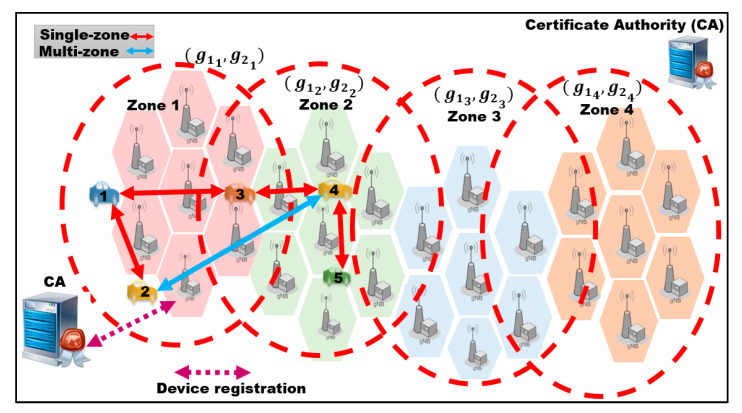
Single-zone and multi-zone communication in the 5G-V2X network model time for the three proposed authentication methods, and six previous related methods for single-zone and multi-zone scenarios.

**Figure 3:**
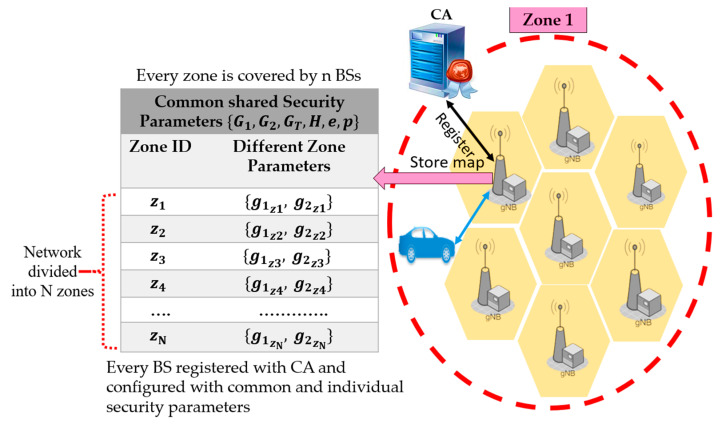
BSs registration with CA.

The deployment of 5G infrastructure requires many Base Stations (BSs), due to its shorter communication range than 4G. For network management purposes, the 5G standard uses a notion of a zone, which is a group of a few BSs. Therefore, we assume that the 5G-V2X network is divided into N zones, where each zone consists of a few BSs, as shown in Figure 2. Our proposed system model includes the certificate authority (CA) that offers security services for the BSs and vehicles. CA has a map for the surrounding road and the positions of BSs on the road. CA initializes security parameters for the BSs in each zone, as shown in Figure 3. Each BS stores the security parameters that are configured by the CA. When entering a zone, each vehicle connects to the nearest BS and requests the key material and authentication parameters for the current zone. Vehicles can communicate in two modes. The first mode is single-zone communication that allows vehicles to securely communicate with other vehicles in the same zone (e.g., in Figure 2, $V_{1}$ in zone 1 communicates with $V_{3}$). The second mode is the multi-zone communication that allows vehicles in one zone to securely communicate with other vehicles positioned in other neighboring zones (e.g., in Figure 2, $V_{2}$ in zone 1 communicates with $V_{4}$ in zone 2). In each zone, all vehicles are configured with common security parameters. However, the vehicles also receive messages from the neighboring zones and thus require the security parameters of the neighboring zones for authentication.

### 3.2. System Initialization

In this section, we discuss the vehicle registration process and the dynamic key generation, based on the elliptical curve cryptography (ECC) bilinear pairing technique.

#### 3.2.1. Vehicle Registration

Vehicles in each zone have a unique pair of secret and public keys to communicate securely with vehicles in the same zone or another zone. A vehicle can send a message to vehicles using a single key pair or a set of key pairs, depending on the selected authentication method. Each vehicle requests the security parameters in advance, including the key pairs for multiple zones that are near the vehicle or are in its travel direction. BSs in each zone provides a security parameters list (secret keys, public keys, zone generators, and pseudo-identities) to the vehicles entering the zone. When a vehicle enters a new zone, it connects directly with the nearest base station and requests the authentication parameters for the current and neighboring zones. The process of authorizing the vehicles to access a 5G cell or zone is conducted using the AKA protocol of 5G standards. The details of the AKA protocol is out of scope for this study, but can be found in [29]. For example, vehicle $v_{i}$ sends an authorization request to the nearest BS, which might include a list of neighboring zone IDs $L_{ZID}$. The BS forwards the authorization request message to the 5G core network, and sends back a response to the vehicle, once the authorization is successful. For each vehicle $v_{i}$, BS generates a list of pseudo identities $Lpid_{i}=\left(pid_{i}{}_{1},\;pid_{i}{}_{2},\;pid_{i}{}_{3}\ldots ,pid_{i}{}_{n}\right),\;$list of secret keys$\;Lsk_{i}=\left(sk_{i}{}_{1},\;sk_{i}{}_{2},\;sk_{i}{}_{3},\ldots ,sk_{i}{}_{n}\right)$, and a list of the corresponding public keys $Lpk_{i}=\left(pk_{i}{}_{1},pk_{i}{}_{2},pk_{i}{}_{3},\ldots ,pk_{i}{}_{n}\right)$, for every zone in the zone ID list $L_{ZID}$, using the zone security parameters. Pseudo identities are used to preserve privacy and allow vehicles to communicate anonymously without exposing their real identities. These pseudo-identities can hide the real identity of the vehicle from other vehicles and prevent tracking attacks. BSs generate a list of pseudo-identities per vehicle, to reduce the vehicles’ frequent communication with the BSs. Pseudo-identities are short IDs that represent the anonymous identity of each vehicle and can replace the full certificate in PKI traditional solutions. We target a certificateless security solution that provides identity authentication using short size IDs. In authentication-based certificate solutions, each vehicle transmits a long certificate with each message that introduce long delays and a high communication cost. In contrast, our protocol supports anonymity and identity authentication, using a short size pseudo-identity. Pseudo-identities are generated by the BSs to allow CA to track vehicles under misbehaving conditions. Each vehicle stores a list of pseudo identities that are valid for a short time to support unlinkability and prevent traceability. Linking of pseudo-identities can disclose some information about the vehicle. We recommend changing pseudo-identity every 10 min, to enhance the security level while protecting the real vehicle information.

We also introduce a dynamic key generation by delegating the key generation to the BSs in each zone. In previous certificate-based methods, vehicles use a pair of the secret key and public key for a long time, which exposes the system key attacks. In contrast, our protocol supports updated key generation in each zone, which allows vehicles to use a different pair of secret and public keys for a short time. We provide the key generation based on the bilinear pairing cryptography over the Elliptic Curve. The BSs configured by the CA with commonly shared security parameters (elliptic curve *E*, two groups of points $\{G_{1},G_{2}\}\;$over the E, one-way hash function H, and a bilinear pairing function e).

**Figure 4:**
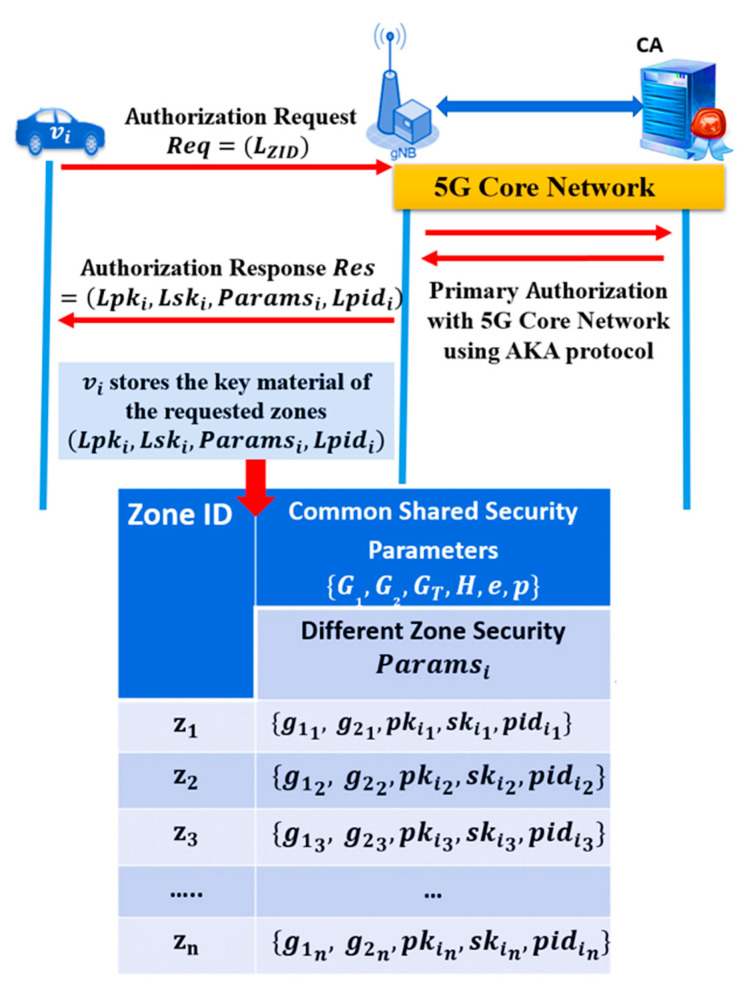
Vehicle primary authorization.

The pseudo-identities, the secret, and public keys are used to support identity authentication and message authentication, respectively. $v_{i}\;$stores $\left(Lpid_{i},Lsk_{i},Lpk_{i}\right)$ until the next security parameters update, as shown in Figure 4.

#### 3.2.2. Dynamic Key Generation

We assume that all zones in the city share the common bilinear pairing parameters $\{G_{1},G_{2},\;G_{T},H,e,p\}\;$described below:

- $G_{1},G_{2}$—two cyclic additive groups of prime order p, based on the elliptic curve E over the finite field$\;F_{p}$ where $G_{1}\times G_{2}$→ $G_{T}$.
- $G_{T}$—a cyclic multiplicative group containing the bilinear pairing result of the two groups $G_{1},G_{2}$.
- H—a cryptographic hash function that maps a message to a point in the group${\;G}_{1}$.
- e—the bilinear pairing function that maps elements from group $G_{1}$ and group $G_{2}$ to group $G_{T}$, as in Equation (1).
- p—a large prime number representing the group order.

Additionally, all zones are configured with individual zone generator parameter $g_{2}{}_{ZID}$.$\;g_{2}{}_{ZID}\;$represents the generator point of the group $G_{2}$ for each zone. The generator point of a group $G_{2}\;$is different for each zone, in order to provide different public keys per vehicle. Each elliptic curve group has a basic point that is used as a generator for all security parameters generated using this group. In our implementation, we used the elements of group$\;G_{2}$ over the elliptic curve, E, to represent the public keys. We configured zones with individual zone$\;g_{2}{}_{ZID}$ to support different public keys. BS picks different random integers to represent the secret keys and use the corresponding$\;g_{2}{}_{ZID}\;$to generate different public keys. Instead of using a single secret and public key for a long time without updating, our protocol allows vehicles to receive different secret and public key in every zone that makes the system resist different key attacks. In the following lines, we describe the key generation method in every zone and the security properties of bilinear pairing cryptography.

- BS picks a random integer$\;sk_{i}\in \;$the finite field $F_{p}\;$that represents a finite element in the range {1 and *p −* 1}.
- BS picks a random integer$\;pid_{i}\in \;$the finite field $F_{p}\;$that represents a finite element in the range {1 and *p −* 1}.
- BS generates a public key for each vehicle in each zone, using the corresponding zone generator and the vehicle assigned secret key: $pk_{i}{}_{ZID}\;=sk_{i}{}_{ZID}.\;g_{2}{}_{ZID}$, where $pk_{i}{}_{ZID}\;\in \;G_{2}$.
- After authorization of a vehicle $v_{i}$ entering a zone, BS sends to $v_{i}$ a message that contains parameters $\left(Lpid_{i},Lpk_{i},Lsk_{i}\right)$ for $v_{i}$, as well as the common parameters $\left\{G_{2},\;G_{T},\;g_{1}{}_{ZID},g_{2}{}_{ZID},H,p,\;e\right\}$ for the zone.

We generate public keys using the group $G_{2}$, then any transmitter can sign a message using its corresponding secret key, to generate a signature that belongs to the group $G_{1}.$ We called this operation bilinear pairing between two groups over the elliptic curve. Using the bilinear pairing between the two groups over the elliptic curve makes the security more complex than the traditional elliptic curve. At the receiver side, the sender’s public key, signature, and the pre-stored zone generator is used to verify the message and accept or reject it. The receiver hash the received message and try to map it to a point in group $G_{1}$, based on the used bilinear pairing function and the zone common parameters. The bilinear pairing-based cryptography relies on the difficulty of the Elliptic Curve Discrete Logarithm Problem (ECDLP).

Before going further to illustrate the bilinear pairing properties, we briefly introduce the ECDLP, and for more information, readers can refer to [30]. Let E be an elliptic curve that is defined over a finite field $F_{p}$, of order p. All points on the elliptic curve E form an additive group usually denoted by $E\left(F_{p}\;\right).\;$In [31], Miller proposed a cryptosystem using a group of elliptic curve points defined over a finite field $F_{p}$. The security level of this cryptosystem relies on the fact that the discrete logarithm problem over this defined group was shown to be hard to solve. This meant that cryptosystems that are designed based on the defined additive groups could achieve a higher or equal level of security with a smaller size for the used secret keys, as compared to other cryptosystems based on different arbitrary groups.

Let G be a finite cyclic group with a generator point g. Given a point a ∈ G, then a = gr for some secret r. Find $r\;=log_{g}\left(a\right)$. In cryptosystems based on the discrete logarithm, the problem that is required to break and solve the system is defined as the Computational Diffie-Hellman Problem (CDHP).

In other words, Elliptic Curve Discrete Logarithm Problem (ECDLP)) can be defined as follows. Given Q, P ∈ E, find an integer a ∈ $F_{p}$ such that P = a, there is no efficient algorithm that can obtain 𝑎 in a short time. Up to now, there is no polynomial algorithm that can solve the ECDLP problem. We briefly introduce the bilinear pairing function e properties using Equations (1)–(4).

Each operation for computing e (P, Q) is a pairing operation where $P\in G_{1}$, $Q\in \;G_{2}$ and $a,\;b\in F_{p}\;$(finite field) [32].

In Equations (1)–(4), e is a pairing function that efficiently satisfies bilinearity, non-degeneracy, and computable properties.

(1) $$e:\;G_{2}\;\times \;G_{1}\to \;G_{T}$$

(2) $$\begin{array}{l} e\;\left(aP,\;Q\right)=\;e\;\left(P\;+\;P\;+\;\ldots \;+\;P,\;Q\right)\\ =e\;\left(P,\;Q\right).\;\ldots \;.e\;\left(P,\;Q\right)\\ =e\;{\left(P,\;Q\right)}^{a}\\ =e\;\left(P,\;aQ\right) \end{array}$$

(3) $$e\;\left(P^{a},\;Q^{b}\right)\;=\;e\;{\left(P,\;Q\right)}^{ab}$$

(4) e (P, Q) ≠1

### 3.3. Three Proposed Authentication Methods

In this study, we propose a lightweight multi-zone authentication protocol that utilizes the bilinear pairing cryptography in message signing and verification. The zoning concept allows vehicles to have security parameters for every zone, without the need for high-cost re-authentication, every time the vehicle moves from one cell to another. The proposed protocol reduces the frequency of key request messages to BSs, by allowing vehicles to request all destination zone parameters in advance.

We propose three authentication methods that utilize ECC pairing-friendly curves to support broadcast multi-zone authentication. The proposed authentication methods are Transmitter Centric Authentication (TCA), Signature Concatenation-Authentication (SCA), and Receiver Centric Authentication (RCA).

#### 3.3.1. Transmitter Centric Authentication (TCA)

In the TCA authentication method, transmitter vehicles generate signatures using their current zone parameters. The receivers in the transmitter’s zone or other zones use the security parameters of the transmitter’s zone to verify the signatures. While the receivers in the same transmitter zone use their pre-stored zone parameters to verify messages, the receivers in other zones search their security parameter table for the transmitter’s zone parameters. If the transmitter’s zone parameters do not exist in the table, the receivers request them from the nearest BS, through a secure channel. The TCA method can also be applied to a single-zone communication, where transmitters and receivers belong to the same zone. In the following, we first describe a single-zone case, followed by a multi-zone case.

**Figure 5:**
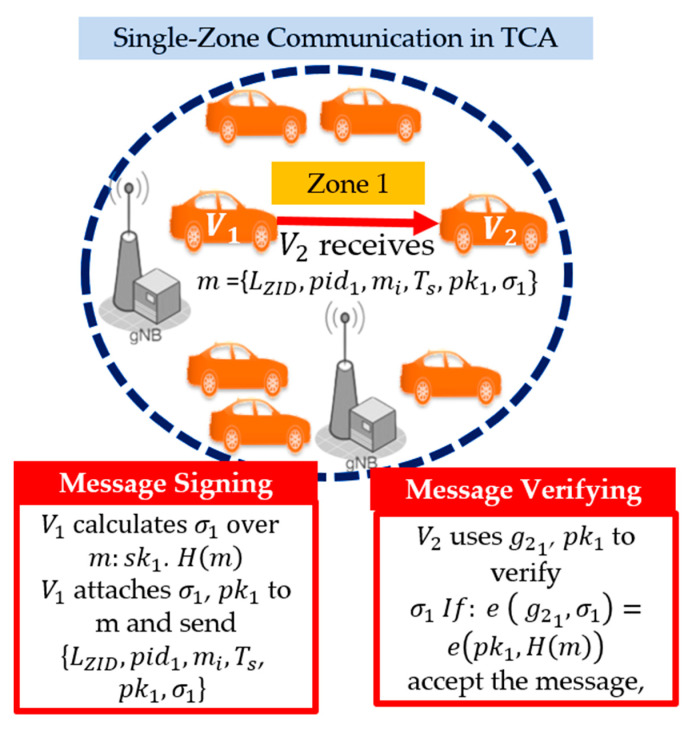
Single-zone communication in the TCA method.

Single-Zone Case: A sender vehicle $V_{1}$ authenticates message m by calculating a bilinear pairing signature $\sigma_{1}$ that can be verified by the receiver vehicle $V_{2}.$ $V_{1}$ computes a hashed message H(m), where m = {$L_{ZID},\;pid_{1},m_{i},T_{s}\}.$ In which $L_{ZID}$ represents a list of zone IDs, $pid_{i}$ represents the pseudo-identity of $V_{1}$, $m_{i}$ represents the message payload, and $T_{s}$ is a timestamp. Then, the hashed message H(m) is mapped to a point in the bilinear group$\;G_{1}$ using its secret key$\;sk_{1}$ in zone 1. Then, $V_{1}$ attaches to each transmitted message the following resulting information—the signature$\;\sigma_{1}$, the public key$\;pk_{1}$, the sender’s pseudo-identity$\;pid_{1}$, zone ID ($z_{1}$), and the current time stamp $T_{s}$, which are illustrated in Figure 5. When receiver $V_{2}$ receives a signed message that contains $\left\{L_{ZID},pid_{1},m_{i},T_{s},pk_{1},\sigma_{1}\right\},\;V_{2}$ checks the freshness of timestamp Ts. If $T_{s}$ is invalid, $V_{2}$ rejects the message; otherwise, $V_{2}$ checks the list of zone IDs $L_{ZID}$. If the receiver’s zone ID matches the sender’s zone ID ($z_{1}$), receiver $V_{2}\;$starts verification of the signature using $pk_{1}\;G_{2},\sigma_{1},\;G_{1}$, and $\;g_{2}{}_{1}$. Receivers accept the message if Equation (5) holds. Otherwise, they reject it.

**Figure 6:**
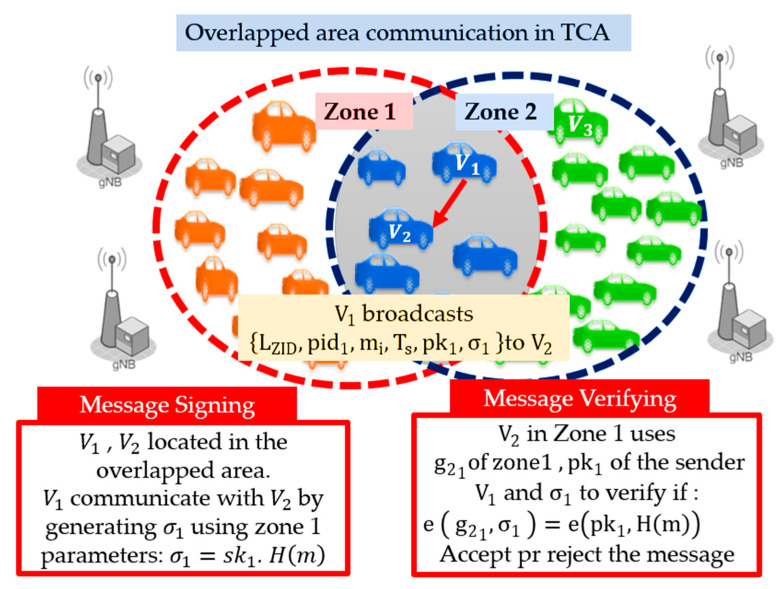
Overlapped area communication in TCA method.

For the transmitter and receiver vehicles located in the overlapped area, they are considered as single-zone communication. TCA algorithm allows vehicles to choose the most updated zone parameters to communicate securely. If the security parameters are updated, all vehicles in the overlapped area receive the updates at the same time. Thus, the vehicles are free to choose the zone parameters, they can use zone 1 information or zone 2 information. As shown in Figure 6,$\;V_{1}$ and $V_{2}$ are located in the overlapped area where $V_{1}$ uses zone 1 security parameters to generate the signature $\sigma_{1}$ over message m. $V_{2}$ received m, checks the freshness of $T_{s}$, if its valid,$\;V_{2}$ checks the list of zone IDs $L_{ZID.}$ The zone ID of the transmitter $V_{1}$ matches the zone ID of the receiver $V_{2}$, $V_{2}\;$starts verification of the signature using $pk_{1}\;G_{2},\sigma_{1},\;G_{1}$, and$\;g_{2}{}_{1}$. Receivers accept the message if Equation (5) holds. Otherwise, they reject it, as shown in Figure 6.

**Figure 7:**
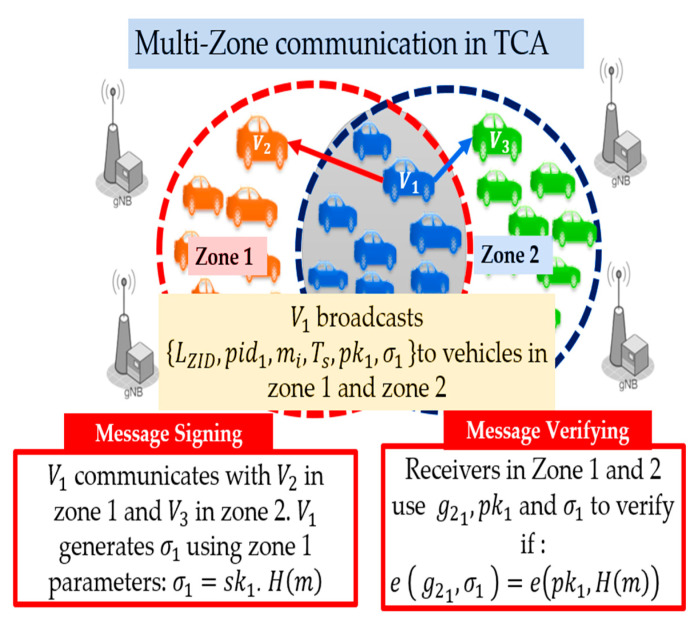
Multi-zone communication in TCA.

Multi-Zone Case: The multi-zone communication of the TCA method is shown in Figure 7. Here, transmitter $V_{1}$ joins zone 1 and zone 2, and thus $V_{1}$ simultaneously transmits to $V_{2}\;$in zone 1 and $V_{3}$ in zone 2. In the TCA method, $V_{1}$ signs the message using zone 1 parameters, while the receivers $V_{1}$ in zone 1 and $V_{2}$ in zone 2 verify using the same parameters as the transmitter.

Receivers in the same zone as transmitter $V_{1}$ use their zone information to verify the signature, while receivers in different zones search the zone parameter storage to find the transmitter’s zone information. If the information is not found, the receiver requests the transmitter’s zone parameters from the nearest BS. Algorithm 1 illustrates the signature generation and verification procedure of the proposed TCA authentication method. The signature verification depends on the bilinear pairing algebraic properties described in Equations (2)–(4). To verify a message under given $pk_{i},\sigma_{i}$, it checks if Equation (5) is held.
**Algorithm 1:** Transmitter-Centric Authentication (TCA) Method**Scenarios:** -One transmitter to many receivers in the same zone (single-zone) -One transmitter to many receivers in different zones (multi-zone) **Signature Generation:**Prepare a message m = {$L_{ZID},\;pid_{i},m_{i},T_{s}\}$Generate a signature$\;\sigma_{i}$ over hashed message *H (m)* using secret $sk_{i}$:$\sigma_{i}=sk_{i\;}.H\left(m\right)$Broadcast $\left\{L_{ZID},pid_{i},m_{i},T_{s},pk_{i},\sigma_{i}\right\}\;$to all zones**Signature Verification:**All receivers use the transmitter zone parameters $\left(\;g_{2}{}_{ZID}\right)\;$to verify $\sigma_{i}$ over m = $\left\{L_{ZID},pid_{i},m_{i},T_{s},pk_{i},\sigma_{i}\right\}$Check $\mathrm{If}\;e\;\left(\;g_{2}{}_{ZID},\sigma_{i}\;\right)=e\left(pk_{i},\;H\left(m\right)\right),$ accept the message, else reject the message.

(5) $$e\;\left(\;g_{2}{}_{ZID},\sigma_{i}\right)=e\left(pk_{i},\;H\left(m\right)\right)$$

If Equation (5) is satisfied, the receiver accepts the message, otherwise, it rejects it. The proof of bilinear pairing verification can be given by Equation (6):

(6) $$e\;\left(\;g_{2}{}_{ZID},\sigma_{i}\right)=e\;\left(\;g_{2}{}_{ZID},sk_{i\;}.H\left(m\right)\right)\;\;=e\;\left(\;sk_{i}.g_{2}{}_{ZID},H\left(m\right)\right)\;=e\left(pk_{i},\;H\left(m\right)\right)$$

Here,

(7) $$sk_{i}.g_{2}{}_{ZID}=pk_{i}$$

In the TCA method, the transmitter vehicle uses the current zone parameters and allows receivers to find the correct zone parameters required to verify the message. The TCA method can allow vehicles in a boundary area to continue using the old zone security parameters. However, the zone parameters might be updated individually, making the verification process for receivers invalid, if some receivers are not updated in a timely manner with the transmitter’s parameter. The next proposed method can provide an alternative solution to this problem.

#### 3.3.2. Signature-Concatenation Authentication (SCA)

**Figure 8:**
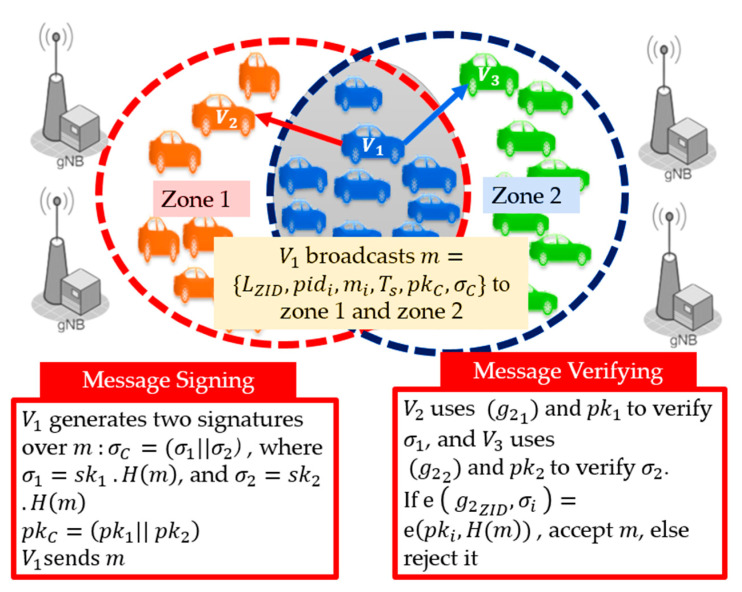
Multi-zone authentication using the signature concatenation (SCA) method.

We introduce the second proposed method, Signature-Concatenation Authentication (SCA), using the example of Figure 8. We propose the Signature-Concatenation Authentication (SCA) method calculates the individual signatures for the receivers that belong to different zones and concatenates the signatures into one. For the case where the receivers are located in N different zones, the transmitter vehicle attaches to its message a concatenated signature of N different signatures, calculated for each zone. Then, the receivers in each zone verify only their signature corresponding to its zone, among the N signatures. The transmitter generates N signatures using the pre-stored secret keys of the transmitter within these communicated neighbor zones and attaches its corresponding public keys for verification. The transmitter provides the zone ID list $L_{ZID}$, which indicates the ordering of the receiver zones to inform each receiver the signature that it should verify among the concatenated signatures. Each receiver verifies only the signature corresponding to their zone ID, using each receiver zone information, as shown in Figure 8.

Algorithm 2 illustrates the authentication procedure of SCA for the example of Figure 6. Vehicles in zone 1 substitute$\;({g_{2}}_{1})\;and\;pk_{1\;}$in Equation (5), to verify $\sigma_{1}\;$over message m, while vehicles in zone 2 verify $\;\sigma_{2}\;$using $\;({g_{2}}_{2})$ and $pk_{2\;}$.This method suffers from a high communication overhead in high-density scenarios where the target receivers are located in multiple zones.

**Algorithm 2:** Signature-Concatenation based Authentication (SCA)
**Signature Generation:**
Prepare message m= {$L_{ZID},\;pid_{i},m_{i},T_{s}\}$Generate$\;\sigma_{1}=sk_{1}.H\left(m\right),\;\sigma_{2}=sk_{2}.H\left(m\right)$,… $\sigma_{N}=sk_{N}.H\left(m\right)$Concatenate the signatures, and their corresponding public keys: $\sigma_{C}=\left(\sigma_{1}||\;\sigma_{2},\ldots ||\sigma_{N}\right),pk_{C}=\left(pk_{1}||\;pk_{2},\ldots ||pk_{N}\right)$,where || represents the concatenation operation of two elements.$v_{i}\;$broadcasts$\;\left\{L_{ZID},\;pid_{i},m_{i},T_{s},pk_{C},\sigma_{C}\right\}$
**Signature Verification:**
All receivers in neighboring zones receive$\;\left\{L_{ZID},\;pid_{i},m_{i},T_{s},pk_{C},\sigma_{C}\right\}$Each receiver checks the $L_{ZID}$ to find its corresponding signature in $\sigma_{C}$ and corresponding public key in $pk_{C}.$Then the receivers use the corresponding $\;g_{2}{}_{ZID}$ to verify. $\mathrm{If}\;e\;\left(\;g_{2}{}_{ZID},\sigma_{i}\;\right)=e\left(pk_{i},H\left(m\right)\right)$ accept the message, else reject the message.


#### 3.3.3. Receiver Centric Authentication (RCA)

Next, we present the third method, Receiver Centric Authentication (RCA). It is a light-weight authentication method that allows vehicles in different zones to communicate using a short aggregated single signature that can be verified by any receiver vehicle that belongs to multiple neighbor zones. In this method, the transmitter vehicle aggregates the generator values of neighbor zones to generate aggregated public keys that allow the receivers to verify the message. In this method, receivers aggregate the neighboring zone’s generators to verify the message. For example, $V_{1}$ in zone 1 received messages from $V_{2}$ in zone 1 and $V_{3}$ in zone 2. Instead of broadcasting one message carrying two signatures for the receivers in the two zones like in the conventional method, RCA generates one message with a single signature to be verified by all vehicles in both zones. Vehicles generate a new aggregated secret key from their original zone’s secret keys to hide the original secrets and increase the security level. The transmitter vehicle searches the security parameters in its pre-stored table for the required$\;(g_{2}{}_{ZID})$. If the parameter cannot be found, it requests the possible combinations of$\;(g_{2}{}_{ZID})$ from the nearest BS. It generates an aggregated public key corresponding to the secret keys {$sk_{1}+sk_{2}+\ldots sk_{N}\}$ for the N neighboring zones that have receivers. The generation of an aggregated public key and aggregated generators for the neighboring zones can be done offline, to reduce the computation time in the vehicles for every transmission. In the RCA method, the transmitter vehicles generate an aggregated secret key$\;sk_{aggr}=sk_{1}+sk_{2}+\ldots sk_{N}$ using the pre-stored vehicle secret keys for the neighboring zones and their corresponding aggregated public key$\;pk_{aggr}=\;sk_{aggr}.\;g_{2}{}_{aggr}$, where$\;g_{2}{}_{aggr}\;={g_{2}}_{1}+\;{g_{2}}_{2}+\ldots +\;{g_{2}}_{N}$ represents the aggregated generators. The transmitter generates message m = {$L_{ZID},\;pid_{i},m_{i},T_{s}\}$, and signs it using the aggregated secret key for the neighboring zones. It then generates a single signature$\;\sigma_{aggr}\;=sk_{aggr}\;.H\left(m\right)$. The transmitter broadcasts $\left\{L_{ZID},\;pid_{i},m_{i},T_{s},pk_{aggr},\sigma_{aggr}\;\right\}$ to all receivers in the neighboring zones. The receivers aggregate the required generators of the neighboring zones as the start of the verification process. Given $g_{2}{}_{aggr},\;pk_{aggr}\in G_{2}$, and $\sigma_{aggr}\in G_{1}$, the receivers verify the message by checking if Equation (8) holds.

(8) $$e\left(g_{2}{}_{aggr},\sigma_{aggr}\;\right)=e\left(\;pk_{aggr},\;H\left(m\right)\right)$$

If Equation (8) is satisfied, the receiver accepts the message, else it rejects the message. Equations (9)–(11) define$\;g_{2}{}_{aggr},\;pk_{aggr}$, and $sk_{aggr}$.

(9) $$g_{2}{}_{aggr}=\;g_{2}{}_{1}+\;g_{2}{}_{2}\ldots +\;g_{2}{}_{N}$$

(10) $$pk_{aggr}=sk_{aggr}\;.g_{2}{}_{aggr}$$

where

(11) $$sk_{aggr}=\;sk_{1}+sk_{2}+\ldots +sk_{N}$$

(12) $$\sigma_{aggr}=sk_{aggr}\;.H\left(m\right)$$

**Figure 9:**
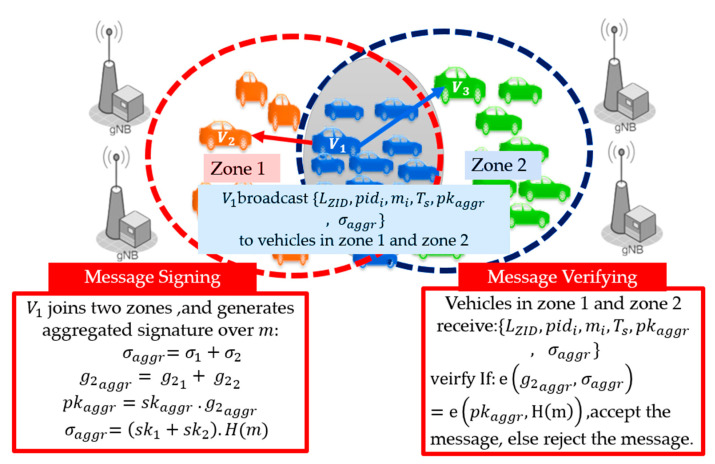
Multi-zone authentication using Receiver-Centric Authentication (RCA) method.

Algorithm 3 summarizes the signature generation and verification procedure of the proposed RCA method. Figure 9 illustrates an example of the RCA authentication method for the two zones. In Figure 7, the vehicles in orange belong to zone 1, the vehicles in green belong to zone 2, and the vehicles in blue belong to zone 1 and 2. Transmitter $V_{1}$ broadcasts a message m to the orange and green receivers, simultaneously. $V_{1}$ signs a message m in the following steps. $V_{1}$ calculates $sk_{aggr}\;=\;sk_{1}+sk_{2}$ followed by $pk_{aggr}=sk_{aggr}.\;g_{2}{}_{aggr}$. $V_{1}$ attaches an aggregated signature $\sigma_{aggr}=\sigma_{1}+\sigma_{2}=sk_{aggr}\;.H\left(m\right)$ to the message. Then, the receivers in zone 1 and zone 2 can verify $\sigma_{aggr}$. The proof of the verification can be proved as follows, in Equation (13):

(13) $$e\left(g_{2}{}_{aggr},\sigma_{aggr}\right)=e\left(g_{2}{}_{aggr},sk_{aggr}\;.H\left(m\right)\;\right)\;=e\left(sk_{aggr}.\;g_{2}{}_{aggr},\;H\left(m\right)\right)\;=e\left(pk_{aggr},\;H\left(m\right)\right)$$

**Algorithm 3:** Receiver-Centric Authentication (RCA)**Signature Generation:**Picks $\;g_{2}{}_{ZID}$ values for the neighboring communicated zonesGenerate $g_{2}{}_{aggr}=\;{g_{2}}_{1}+\;{g_{2}}_{2}+\ldots +\;{g_{2}}_{N}\in G_{2}$$sk_{aggr}=sk_{1}+sk_{2}+\ldots +sk_{N}$$\in \;F_{p}$$pk_{aggr}=sk_{aggr}\;.g_{2}{}_{aggr},$ where $pk_{aggr}$$\in G_{2}\;$Generate$\;\sigma_{aggr}=sk_{aggr}\;.H\left(m\right)$, where $\sigma_{aggr}\in G_{1}$$v_{i}$ broadcasts$\;\left\{L_{ZID},\;pid_{i},m_{i},T_{s},pk_{aggr},\sigma_{aggr}\;\right\}$**Signature Verification:**All vehicles in the neighboring zones receive $\left\{L_{ZID},\;pid_{i},m_{i},T_{s},pk_{aggr},\sigma_{aggr}\;\right\}\;$Receivers generate $g_{2}{}_{aggr}$ for the neighboring zones, using $g_{2}{}_{aggr},\sigma_{aggr},\mathrm{and}\;pk_{aggr}\;$to verify:$If\;e\left(g_{2}{}_{aggr},\sigma_{aggr}\;\right)=e\left(\;pk_{aggr},\;H\left(m\right)\right),$ accept the message, else reject the message.

## 4. Security Analysis of the MAPP Protocol

### 4.1. Security Requirement Analysis

Every security protocol must satisfy some primary security functions defined by the V2X security standards. The security requirements that must be satisfied by the proposed protocol is identity authentication, message authentication, non-repudiation, privacy-preserving, unlinkability, and system update [33].

In this section, we show how the proposed MAPP protocol accomplishes the required security functions.

#### 4.1.1. Identity Authentication

In our protocol, each vehicle is registered with the BS using its real information. BSs are configured with common security parameters and individual security parameters to generate pseudo-identities for the vehicle. Pseudo-identities allow vehicles to communicate without revealing their real identities. Identity authentication is satisfied in the three proposed authentication methods, by allowing the dynamic random numbers to hide the real identity. Each transmitted message in our proposed methods is attached with $pid_{i}$, that is generated by an authorized third party (BSs). Each vehicle receives a list of updated pseudo-identities from the nearest BS, which can be used to authenticate the identity of each vehicle. Under misbehaving conditions, vehicles report the $pid_{i}$ to the BS to remove the malicious vehicle from the network. We called our protocol certificatless bilinear pairing, as we replaced the long certificate in PKI solutions with a short size pseudo-identity that provides identity authentication and anonymity.

#### 4.1.2. Message Authentication

Message authentication represents proof that the message has not been changed during transmission. Our protocol provides message authentication by calculating a short bilinear pairing signature over each transmitted message. The signature calculation is done by hashing the message and mapping it to a point over the elliptic curve, then signing it with the sender’s secret key. Verification of signature at receivers can provide the message authentication. In Equations (5), (7), and (8), each receiver in the different proposed methods can use the corresponding bilinear pairing equation to verify the signature, based on the received public key$\;pk_{i}$, the zone generator$\;g_{2}{}_{ZID}$ and the calculated signature$\;\sigma_{i}$. Verifying the validity of $e\;\left(\;g_{2}{}_{ZID},\sigma_{i}\right)=e\left(pk_{i},\;H\left(m\right)\right)$ proves the message authenticity. If the verification failed at the receiver side, the message must be discarded.

#### 4.1.3. Non-Repudiation

Any proposed security protocol must allow the non-repudiation service by providing the identity of the message sender, the accurate sending time, and the accurate location. The non-repudiation can prevent any sender from denying sending of any malicious message. If this service is not guaranteed, any driver can disseminate malicious messages without any punishment. Our proposed MAPP protocol attaches a pseudo-identity $pid_{i}$ and a timestamp $T_{s}\;$to each message m $=\left\{L_{ZID},\;pid_{i},m_{i},T_{s},pk_{i},\sigma_{i}\right\}$, to prove the non-repudiation requirement.

#### 4.1.4. Privacy-Preserving

Privacy is an important security requirement that should be satisfied by the proposed security protocol. Privacy preservation is satisfied by hiding the real identity of vehicles and providing anonymity using pseudo-identity. In our protocol, we provide anonymity while allowing certificate authority organizations to trace the misbehaving vehicle and revoke them under misbehaving conditions. The trade-off between hiding the real identity and allowing CA to trace vehicles is a critical requirement that is satisfied in our protocol by generating a$\;pid_{i}$ that has a relation with the initial registered information of each vehicle. Under misbehaving conditions, CA maps the vehicle’s pseudo-identity. In our proposed protocol, we assume that the pseudo-identity consists of two parts, the first part is $pid_{1}=h\left(Ri\right)$, where Ri is a dynamic random number Ri, the second part is fixed $pid_{2}=\;pid_{init}⊕h\;\left(pid_{1}\right)$, represented by the XORing between the initial pseudo-identity $pid_{init}\;$and the hashed value of $pid_{1}.$ $pid_{init}$ reflects the real identity of each vehicle. The $pid_{i}=pid_{1}||pid_{2}\;$can preserve privacy while allowing the traceability of vehicles by the CA, under misbehaving conditions.

#### 4.1.5. Unlinkability

The proposed methods use pseudo-identity change to make it hard for an attacker to link the new pseudo-identity with the old one, which preserves both the identity and the location privacy. The previous V2X standards suffer from position tracking problems. Our methods, however, never disclose the vehicle’s real identity, as the real identity is stored securely in the CA. Each pseudo-identity is composed of two parts—the first dynamic part is $pid_{1}=h\left(Ri\right)$ with a random number Ri that changes with every transmitted message The second part is $pid_{2}\;$where a fixed value $pid_{init}$ allows the CA to track the malicious vehicle, while the full pseudo-identity $pid_{i}$ makes it hard to link two pseudo-identities.

#### 4.1.6. System Updates

Our protocol provides a dynamic key generation in each zone that allows vehicles to use different secret and public keys for a short time. System updates prevent the key compromising attacks and protect the security material from sniffing attacks. Using a single key for a long time can be hacked within a defined time, after many trials to break it. Our system supports a list of secret keys, public keys, and pseudo-identities that help the vehicles to use short time keys and pseudo-identities.

From the previous analysis of different security requirements, we can prove that the proposed authentication methods support the standard security requirements, with a low-cost overhead.

### 4.2. Resistance to Attacks

The proposed protocol is secure against some common attacks described below.

#### 4.2.1. Replay Attack

The proposed authentication method ensures the freshness of the transmitted message by attaching the current timestamp. For example, in the TCA method, the message format is $\left\{L_{ZID},pid_{1},m_{i},T_{s},pk_{i},\sigma_{i}\right\}$, where Ts is the attached time stamp. All vehicles should be synchronized to provide accurate time and resistance against the replay attacks. The synchronization of vehicles can be provided using GPS devices.

#### 4.2.2. Modification Attack

In our protocol, message integrity is achieved using a short signature generated using an elliptic curve. The sender generates a message m, then calculates a signature $\sigma_{i}$ over m by hashing m and then mapping it to a point on the elliptic curve using the secret key $sk_{i}$. The sender attaches the signature and the public key $pk_{i}$ to allow the receiver to calculate a signature over the received message and then compare the transmitted signature and calculated signature to accept or reject the message. If the verification of $\left(g_{2}{}_{ZID},\sigma_{i}\right)=e\left(pk_{i},\;H\left(m\right)\right)$ is true, the receiver accepts the message, else it drops it. In this way, by verifying the signature over each message, our protocol ensures the message integrity and prevents-message alteration that proves our protocol’s ability to resist modification attacks.

#### 4.2.3. System Key Compromising Attacks

In our protocol, we use a certificataless public key authentication algorithm that supports the high-security level, using a pair of secret keys and public keys. In contrast to protocols based on a single secret key that sends the shared secret for each message, to allow message verification at the receiver side. In our methods, we send the public key to allow the receiver to authenticate the signature that generated the message, using the corresponding secret key. For the RCA method, we generated a new secret key from the original secrets and for the communicated zones and a new public key using the zone generators of the communicated overlapped zones. Hiding the original secret keys, enhances the security level of the RCA method over the TCA and SCA method. Only authorized vehicles that registered with the BSs have access to the security zone parameters ($g_{2}{}_{ZID})$. All vehicles at the initialization step register with the BS to receive the zone security parameters, after this, all parameters are stored securely at vehicles. Our protocol did not transmit the individual security parameters that make the system security high and difficult to break.

#### 4.2.4. DOS Attacks

Our three proposed authentication methods support the immediate verification of packets. In contrast to key disclosure protocols that allow the receiver to wait till the sender discloses the signing key. The key disclosure protocols allow receivers to buffer a high number of packets until the key is received. Overwhelming the receiver buffer prevents the receiver from verifying the packets and result in message loss. For this, a large buffer size is required for key disclosure protocols, while in our authentication method, a small buffer is required to store a list of pseudo-identities, secret keys, and public keys $\left(Lpid_{i},Lsk_{i},Lpk_{i}\right)$.

From the previous analysis, we can summarize that our protocol can satisfy a wide range of security analysis and can resist a different type of attacks.

## 5. Communication Overhead Analysis

In this section, we compare the proposed MAPP protocol and the certificateless bilinear pairing methods [19,20,21,22,23,24]. To calculate the communication cost, we analyze the message structure of the MAPP protocol and the previous methods, based on the bilinear pairing [19,20,21,22,23,24]. For the security overhead calculations, we exclude the size of the traffic message payload, since it is common for all methods. In our implementation, we define the elliptic curve equation E over a finite field $F_{p}$, which is given by Equation (14).

(14) $$y^{2}=x^{3}+b\;mod\;p$$

We employ the Barreto–Naehrig (BN256) curves that offer asymmetric bilinear pairing (e.g., $G_{1}\;$≠ $G_{2}$) [34]. For BN256, we choose r = 256 bits to give finite field points $2^{256}$ and b a random number ≠ 0 to be a non-singular curve, which means that the curve has no cusps or self-intersections. The BN curve chooses b = 2 to satisfy the non-singular condition and give optimum security. $G_{1}$ indicates the cyclic additive subgroup defined over $F_{p}$,$G_{2}$, denoting a cyclic additive subgroup defined over $F_{p2}$, and $G_{T}$ represents the cyclic multiplicative subgroup defined over$\;F_{p12}$. $G_{1}$, $G_{2}$, and $G_{T}$ are defined with order r. $G_{1}$ and $G_{2}$ elements are represented in a compressed form by the values of the x-coordinate instead of representing them by (x, y, z), which reduces their sizes to 32 and 64 bytes, respectively [35]. Table 3 summarizes the BN256 curve’s parameters and element size in the bilinear groups.

In the following, we analyze the overhead message of six previous methods (1) Horng et al. [19], (2) Li et al. [20], (3) Malhi et al. [21], (4) Lin et al. [22], (5) Bayat et al. [23], and (6) Boneh et al. [24], as well as the proposed MAPP.

(1) Horng et al. [19]:

The structure of the transmitted message is expressed by Equation (15):

(15) $$m=\{M_{i},ID_{i},vpk_{i},t_{i},\sigma_{i\;}\}$$

where$\;M_{i}$ is the message payload, $ID_{i}\;$represents the vehicle pseudo-identity, $vpk_{i}$ represents the vehicle public key,$\;t_{i}$ represents the time stamp, and $\sigma_{i\;}$ represents the signature. $ID_{i}$ consists of two parts $\left\{ID_{i}^{1},ID_{i}^{2}\right\}$, where $ID_{i}^{1}\in \;G_{2}$, $ID_{i}^{2}\in \;F_{p},\;and\;vpk_{i},\;\sigma_{i\;}\in G_{2}$. The total communication overhead for the message of Equation (15) is $|ID_{i}\left|+|vpk_{i}\right|+\left|t_{i}\right|+|\sigma_{i\;}|=$ 64 + 32 + 64 + 4 +64 = 228 bytes.

(2) Li et al. [20]:

The structure of the transmitted message in [20] is also represented by Equation (15) and introduces a total communication overhead of 228 bytes.

(3) Malhi et al. [21]:

The structure of the transmitted message is expressed by Equation (16):

(16) $$m=\{M_{i},PS_{j},PS_{1j},P_{i},t_{i},\sigma_{ijK\;}\}$$

where${\;M}_{i}$ is the message payload, while the vehicle’s pseudo-identity is represented by two parts {${\mathrm{PS}}_{j},{\mathrm{PS}}_{1j}$}$\in G_{2}$. $P_{i}$ represents the public key $\in G_{2}$, and $t_{i}$ represents the time stamp. $\sigma_{\mathrm{ijK}\;}\;$represents the signature over the message and consists of two parts {$U_{i},V_{\mathrm{ijk}}$}$\in G_{2}$. The total communication overhead for the message of Equation (16) is $\left|{\mathrm{PS}}_{j}\left|+\left|{\mathrm{PS}}_{1j}\right|+\left|P_{i}\right|+\left|t_{i}\right|+\right|\sigma_{\mathrm{ijK}\;}\right|$ = 64 + 64 + 64 + 4 + 64 + 64 = 324 bytes.

(4) Lin et al. [22]:

The structure of the transmitted message is defined in Equation (17), where group ID is used to identify the group where the vehicle belongs. The message payload includes information about the vehicle’s position, time of transmission, the direction of travel, and traffic events. A timestamp of 4 bytes is used to prevent the message replay attack. The signature over the message consists of 3 elements of $G_{2}$. The Time-To-Live (*TTL*) controls how long the message is allowed to remain in the network.

(17) $$m=\{L_{payload\;},L_{groupID\;},L_{msgID},L_{timestamp\;},L_{signature\;},L_{TTL}\}$$

The total communication overhead for the message of Equation (18) is

(18) $$|L_{groupID}\left|+|L_{msgID}\right|+|L_{timestamp\;}\left|+\right|L_{signature\;}\left|+|L_{TTL}\right|=2\;+\;2\;+\;4\;+\;3\times 64\;+\;1\;=\;201\;\mathrm{bytes}.$$

(5) Bayat et al. [23]:

The parameters for transmitted messages are represented by Equation (19):

(19) $$m=\left\{M_{i},ID_{i},\sigma {\;}_{i},\;T_{i}\right\}$$

where a message payload $M_{i}$, a pseudo-identity IDi ∈ $G_{2}$ consists of two parts (ID1, ID2), a signature σi ∈ $G_{2}$, and a timestamp Ti. The communication overhead of one message is$\;|ID_{i}\left|+\left|\sigma {\;}_{i}\right|+\right|T_{i}|$ =2×64 + 64 + 4 = 196 bytes.

(6) Boneh et al. [24]:

The parameters for the transmitted message are calculated using Equation (20):

(20) m={M,gpk, σ, T}

where a message payload M, $gpk=\;\left(g_{1},\;g_{2},\;h,\;u,\;v,\;w\right)$ represents the group public key that consists of 5 elements$:\;g_{1}\in G_{1},$ $\;g_{2}\in G_{2},\;\left\{h,\;u,\;v\right\}$ $\in G_{1},$ and $w\;\in G_{2}$. Additionally, a group signature σ consists of three elements of $G_{1}$ and six elements of $F_{p}$, and a timestamp T.

The total communication overhead for the message of Equation (20) is |gpk|+|σ|+|T| = 32 + 64 + (3 × 32) + 64 + (3 × 32) + 6 × 32 + 4 = 548 bytes.

### 5.1. Proposed TCA Method

**Figure 10:**
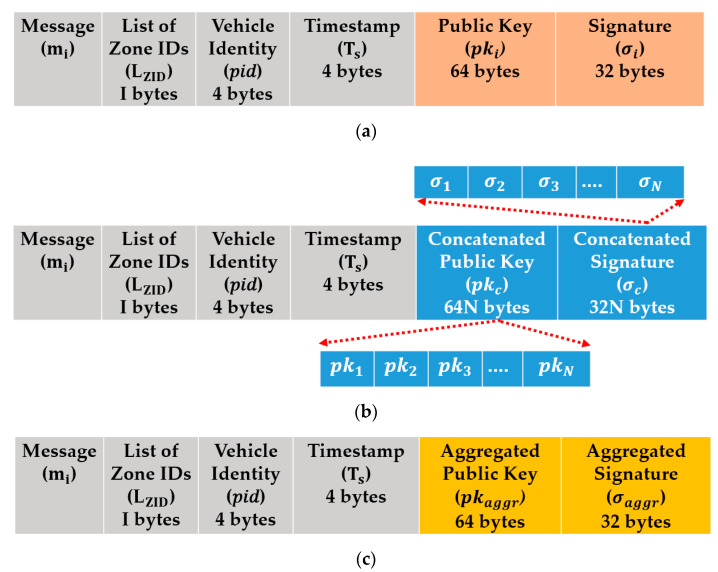
The proposed protocol message structure. (**a**) TCA method, (**b**) SCA method, and (**c**) RCA method. Where $m_{i}$ represents the message payload, $pid_{i}$ represents the pseudo-identity of $v_{i}$, and $L_{ZID}$ represents a list of zone IDs. $T_{s}$ represents the time stamp, $pk_{i}$ represents the public key of $v_{i}$, and $\sigma_{i}$ represents the signature over the message. In our implementation, a signature $\sigma_{i}\in G_{1}$ and the public key $pk_{i}$ is $\in G_{2}$. The total communication overhead of one message is $|L_{ZID}\left|+|pid_{i}\right|+\left|T_{s}\right|+\left|pk_{i}\right|+\left|\sigma_{i}\right|=$ 4 + 4 + 1 + 64 + 32 = 105 bytes.

The message structure of the proposed authentication TCA method is shown in Figure 10a, which is analyzed as follows. In the TCA method for single-zone communication, the transmitted message structure is represented by Equation (21).

(21) $$m=\{m_{i},L_{ZID},pid_{i},T_{s},pk_{i},\sigma_{i}\}$$

### 5.2. Proposed SCA Method

The message structure of the proposed authentication SCA method is shown in Figure 10b, we analyzed the SCA message structure using Equation (22):

(22) $$m=\left\{m_{i},L_{ZID},\;pid_{i},T_{s},pk_{C},\sigma_{C}\right\}$$

where $pk_{c}=N\left|pk_{i}\right|,\;\mathrm{and}\;\sigma_{C}=N|\sigma_{i}|.$ Therefore, the total communication overhead was $|L_{ZID}\left|+|pid_{i}\right|+\left|T_{s}\right|+\left|pk_{c}\right|+\left|\sigma_{c}\right|$4 + 1 + 4 + 64*N* + 32*N* = 9 + 96*N* bytes. The communication overhead for the SCA method depend on the number of communicated zones *N*.

### 5.3. Proposed RCA Method

The message structure of the proposed authentication RCA method is shown in Figure 10c, we analyzed the RCA message structure using Equation (23). RCA method support multi-zone authentication based on the aggregated security parameters of the communicated zones.

(23) $$m=\left\{L_{ZID},\;pid_{i},m_{i},T_{s},pk_{aggr},\sigma_{aggr}\;\right\}$$

In RCA, the total communication overhead is $|L_{ZID}\left|+|pid_{i}\right|+\left|T_{s}\right|+\left|pk_{aggr}\;\right|+\left|\sigma_{aggr}\right|\;$= 1 + 4 + 4 + 64 + 32 = 105 bytes.

In SCA and RCA, we choose 1 byte for $L_{ZID}$, as we assume that the maximum number of zones cannot exceed 256. While in the TCA method, we only send a single transmitter zone ID.

**Figure 11:**
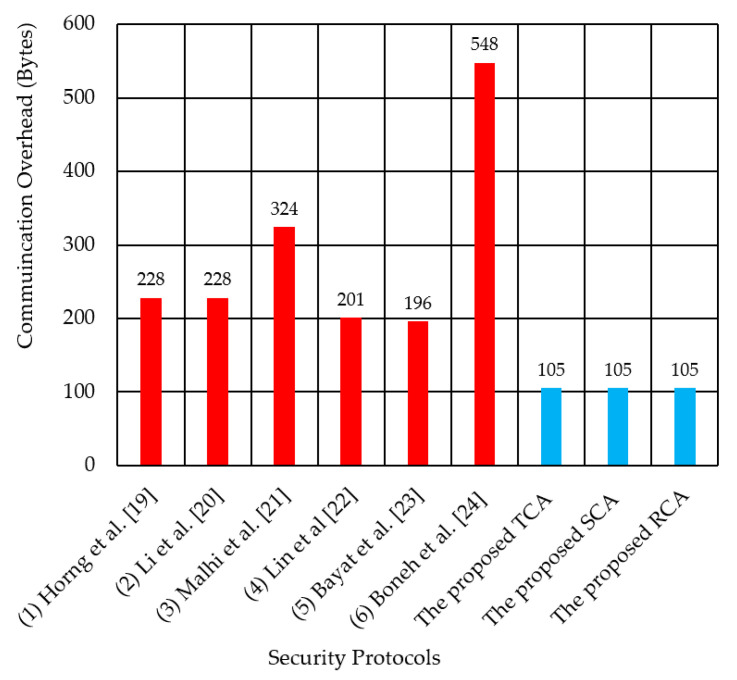
Comparison of communication cost per message for the three proposed authentication methods and the compared security protocols for single-zone scenario.

Figure 11 compares the communication cost of the three proposed methods and the six previous methods in single-zone communication. It shows that the three proposed methods outperform all six previous methods, by reducing the communication cost by 50–80% in a single-zone scenario.

In a multi-zone scenario, all previous methods repeatedly transmit the same message for multiple individual destination zones that require different signing keys. In contrast, the proposed authentication methods (TCA, SCA, RCA) send a single signed message to N zones and allow the receivers in multiple zones to verify the same message.

**Figure 12:**
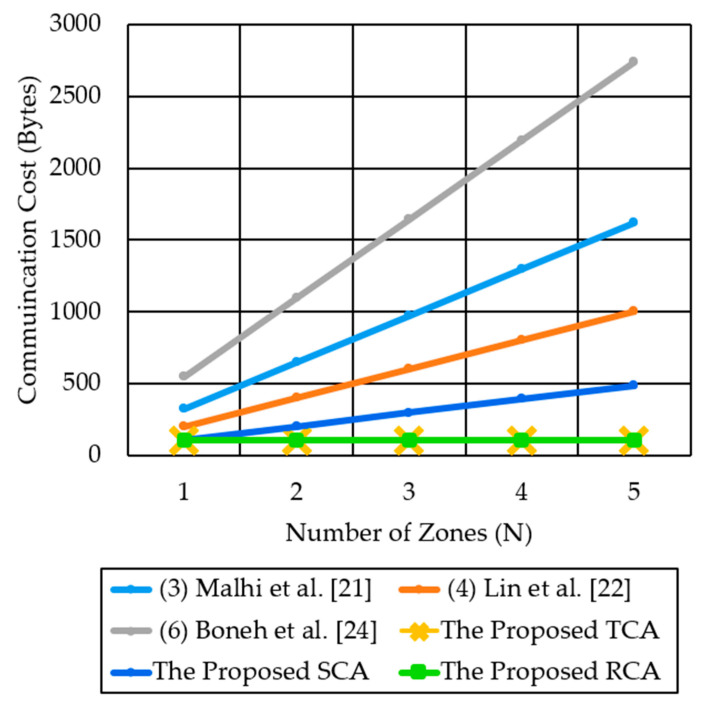
Comparison of communication cost for the proposed authentication methods and the compared security protocols for the multi-zone scenario.

Figure 12 compares the communication cost of the three proposed methods with three previous methods (3) Malhi et al. [21], (4) Lin et al. [22], and (6) Boneh et al. [24]. We choose to compare our methods with the previous [21,22,24], only because we found that the other protocols introduce nearly the same communication overhead. We tried to show the differences between our methods and the previous methods.

TCA and RCA incur a constant communication cost of 105 bytes per message, irrespective of the number of destination zones. In contrast, the proposed SCA method and the previous methods [21,22,24] show the communication cost increasing along with the number of destination zones N. However, the proposed SCA method shows much lower growth in the increase of the cost than the previous methods. For example, in the case of N = 5, SCA has a cost of only 489 bytes, while the previous methods, Boneh et al. [24], Lin et al. [22] and Malhi et al. [21] incur significantly higher communication cost, as high as 2740, 1005, and 1620 bytes, respectively. For multi-zone communication of 5 destination zones, the proposed TCA and RCA methods reduce the communication cost by 26 times, while the SCA method reduces the communication cost by 2–5 times, as compared to the three previous methods of [21,22,24].

## 6. Computation Overhead Analysis

To evaluate the computation overhead of the proposed protocol, we implemented it using a simplified C-V2X protocol simulator and a bilinear pairing cryptography library called MCL [36]. MCL implements functions for the elliptic curve cryptography that supports optimal pairing over (BN) curves. We chose a BN curve with an embedding degree k = 12, which supports a 128-bit security level over a prime field of size 256 bits [37].

To compare the computation overhead, we also implemented the six previous methods [19,20,21,22,23,24] that were analyzed in Section 4. We tested all methods under the same experimental environment for a fair comparison. Table 4 shows the average execution time of the primary cryptographic operations of the BN256 curve in our simulator. The simulations are conducted in a hardware platform employing an Intel Core I7-4770 processor with a 3.6 GHz clock, Linux gcc.5.4.0, and a main memory of 4 GB.

In the following, we analyze the signature generation time and signature verification time of the six previous methods—(1) Horng et al. [19], (2) Li et al. [20], (3) Malhi et al. [21], (4) Lin et al. [22], (5) Bayat et al. [23], and (6) Boneh et al. [24], as well as the proposed MAPP for the single-zone and multi-zone scenarios. We analyze the computation overhead for signature generation and signature verification. In Horng et al.’s protocol [19], the signature generation per message requires two scalar multiplication and two addition in the group$\;G_{1}$, which results in a computation overhead of 2TM_G1_ + 2TA_G1_ = 0.9594 ms. Its signature verification per message requires three pairings, one scalar multiplication in$\;G_{1}$_,_ and one hash map, which results in a computation overhead of 3TP + TM_G1_ + TM2P = 3 × 2.446 + 0.479 + 0.135 = 7.8305 ms.

Similarly, we analyzed the signature generation time and verification time of the other previous methods [20,21,22,23,24]. The analysis results are summarized by the formulas in Table 5.

Finally, we analyzed the signature generation time and verification time for the three proposed MAPP protocol.

### 6.1. Proposed TCA Method

For the TCA method in single-zone communication, signature generation requires one hash computation, mapping to a point in the group$\;G_{1}$ and one scalar multiplication over group$\;G_{1}$, where $\sigma_{i}=sk_{i}\;.H\left(m\right).$ Hence, the signature generation time can be represented by TM2P + TM_G1_ = 0.479 + 0.135 = 0.614 ms. Signature verification requires two pairing operations to check the validity of the bilinear pairing operation, where $e\;\left(\;g_{2}{}_{ZID},\sigma_{i}\right)=e\left(pk_{i},\;H\left(m\right)\right).$ Thus, the signature verification time is two pairing operations and one HashAndMapTo operation = 2TP + TM2P = 4.9 + 0.135 = 5.035 ms. For the case of TCA, both the signature generation and verification overhead is constant, irrespective of the number of destination zones. The total computation time due to security overhead is the combination of signature generation time and signature verification time. For the TCA method, the computation overhead is TM2P + TMG1 +2TP+TM2P = 0.479 + 0.135 + 4.9 + 0.135 = 5.649 ms; (the cryptographic operations used in the computation time calculations are defined in Table 4).

### 6.2. Proposed SCA Method

In the case of the SCA method, the signature generation time of$\;\sigma_{C}=N|\sigma_{i}|$, where$\;\sigma_{C}$ is n concatenated signatures per message that consumes n(TM2P + TMG1) = n (0.479 + 0.135) = 0.614n ms, where a single signature generation time is 0.614ms. On the other hand, the signature verification consumes two pairing operations and one HashAndMapTo operation = 2TP+TM2P = 4.9 + 0.135 = 5.035 ms. Verification time incurs only a constant overhead of 5.035 ms, since the individual receiver verifies only its corresponding signature and ignores the other signatures. We also assume that concatenation operations of n signatures at the transmitter side is neglected, as compared to the signature generation time. We assume that the searching time for the correct zone parameters is negligible, as compared to the verification time. Thus, the total computation time for the SCA method is the concatenated signature generation time, and the individual verification time at each receiver is (0.614n) ms + 5.035 ms. The computation time of SCA depends on the number of destination zones n.

### 6.3. Proposed RCA Method

The computation time at senders including the aggregation time of $sk_{aggr},$ and $pk_{aggr}.$

$sk_{aggr}$ consumes n−1 addition operations over $F_{p}:\;$(n−1)TAFp = 0.001 (n−1)ms. While $pk_{aggr}\;$needs n−1 addition operations in a group $G_{2}$ = (n−1)TAG2 = 0.013 (n−1) ms.

Therefore, the generation time of single signature $\sigma_{aggr}=sk_{aggr}\;.\;H\left(m\right)\;$requires mapping to a point in the group$\;G_{1}$, and one scalar multiplication over $G_{1}$ and the aggregation time of $sk_{aggr},\;$and $pk_{aggr}.\;$Therefore, the signature generation time is TM2P + TMG1 +(n−1 )TAFp+(n−1 )TAG2=0.001 (n−1)+0.013 (n−1) = 0.600 ms + n*0.014 ms.

While the signature verification per message requires the aggregation of $g_{2}{\;}_{aggr}\;$that requires n−1 addition in $G_{2},\;$mapping to a point in the group$\;G_{1}$, and one scalar multiplication over $G_{1}$. Therefore, the signature verification time is (n−1)TAG2 + TM2P + 2TP = 0.013 (n−1)+ 0.135 + 4.9 = 5.014 + n*0.013 ms.

Thus, the total computation time for the RCA method including the signature generation time, the verification time, and the aggregation time of $sk_{aggr},\;pk_{aggr}\;$at the transmitter$,\;\mathrm{and}\;g_{2}{\;}_{aggr}\;\mathrm{time}\;\mathrm{at}\;\mathrm{receiver}\;\mathrm{is}$ 0.600 ms + n*0.014 ms + 5.014 + n*0.013 ms. Therefore, the total computation time is 5.614 ms + (0.027n) ms. The computation time of SCA depends on the number of destination zones n.

From the described analysis, we found that the TCA method computation time is the lowest among the other proposed methods (SCA and RCA). While the RCA method introduces a little overhead, as compared to the high overhead of the SCA method that requires the generation of n signatures at transmitters.

Table 5 compares the computation overhead of the three proposed authentication methods and the six previous methods [19,20,21,22,23,24], for single-zone and multi-zone scenarios. In a multi-zone scenario, the six previous methods incur a high computation overhead to sign n messages for N destination zones. In contrast, the three proposed methods in a multi-zone scenario send a single message with a single short signature.

**Figure 13:**
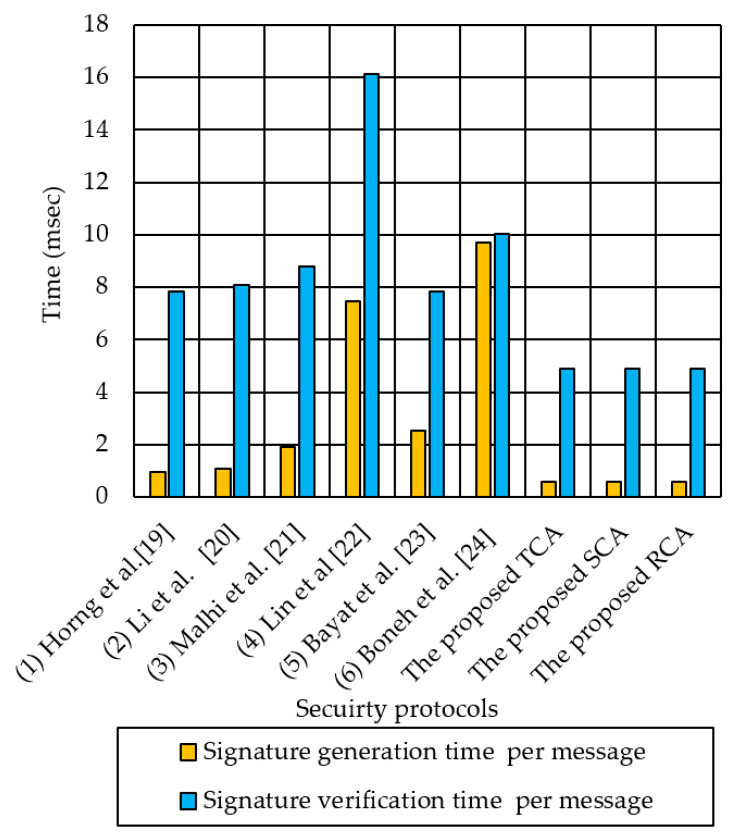
A comparison of signature generation time and verification time for the proposed TCA method and the six previous methods in a single-zone scenario.

Figure 13 shows the computation cost per message, including the signature generation time and verification time for the three proposed authentication methods and the six previous methods [19,20,21,22,23,24], for the single-zone scenario. For the single-zone, the signature generation time per message of Boneh et al. [24] is as long as 9.7275 ms, as one signature generation requires 3 bilinear pairings and five scalar multiplication over group $G_{1}$. It is 16 times longer than the proposed three authentication methods, which consume only 0.614 ms for signature generation. For signature verification, the previous methods [19,20,21,22,23,24] incur as long as 16 ms (for the case of [22]), due to their excessive use of bilinear pairing in verification. It is 4 times longer than the three proposed authentication methods that incur only 5.035 ms for verification.

**Figure 14:**
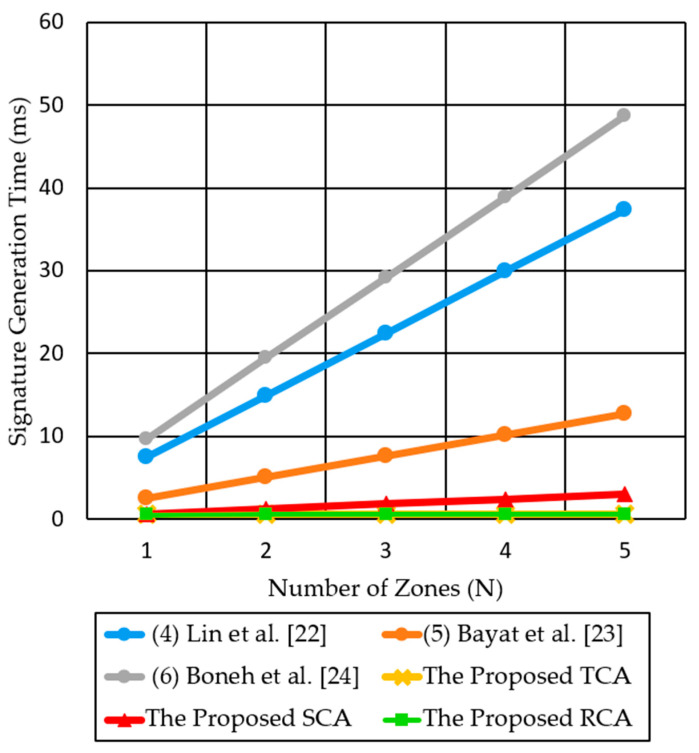
Comparison of signature generation time for the three proposed authentication methods and the two previous methods for multi-zone scenarios.

In a multi-zone scenario, the previous methods repeatedly transmit the same message with different signatures to multiple destination zones. However, the three proposed authentication methods send a single signed message to multiple receivers in different zones. Each receiver in multiple zones individually verifies the same signature by just one verification step, which provides a constant verification time regardless of N. As shown in Figure 14, we compare the signature generation time in multi-zone scenarios for the three proposed authentication methods (TCA, SCA, RCA) and three previous methods, Lin et al. [22], Bayat et al. [23], and Boneh et al. [24]. TCA for multi-zone scenarios introduces a fixed computation time of 0.614 ms for a signature generation, regardless of the number of destination zones. SCA introduces 0.614n ms, which linearly increases with the number of destination zones. While RCA introduces a little increase in singing time, due to aggregation of $sk_{aggr}\;$and $pk_{aggr}$.

For N = 5, Lin et al. [22], Bayat et al. [23], Boneh et al. [24], SCA, TCA, and RCA consume signature generation time of 37.3 ms, 12.745 ms, 48.6 ms, 3.07 ms, 0.614 ms, and 0.67 ms, respectively. Therefore, the proposed methods significantly reduce the signature generation time by 16 times–80 times, as compared to the two previous methods [22,24].

**Figure 15:**
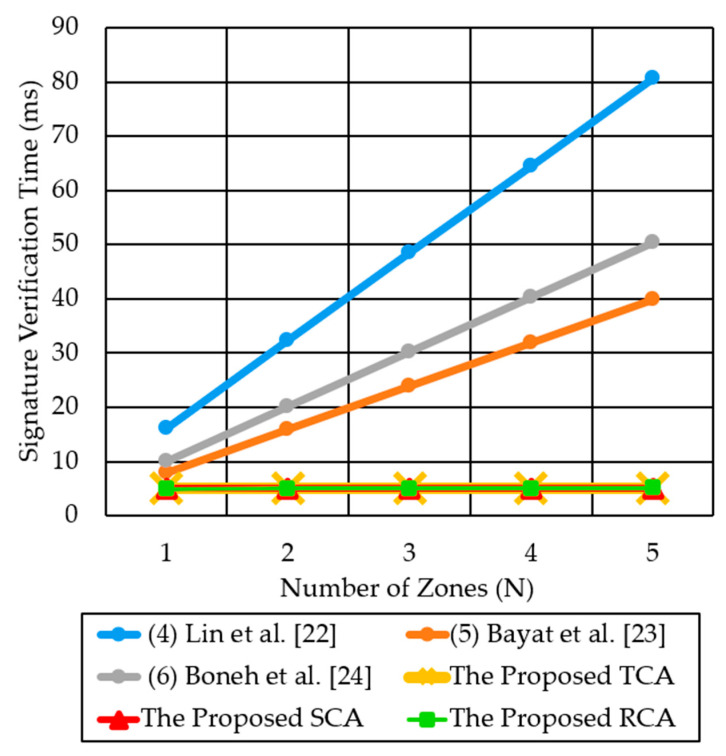
Comparison of signature verification time for the three proposed authentication methods and the two previous methods for multi-zone scenarios.

As shown in Figure 15, we compare the signature verification time in multi-zone scenarios for the three proposed authentication methods (TCA, SCA, RCA) and two previous methods, Lin et al. [22] and Boneh et al. [24]. TCA and SCA for multi-zone scenarios introduce a fixed computation time of 5.035 ms, for a signature verification, regardless of the number of destination zones. For N = 5, Lin et al. [22], Boneh et al. [24], SCA, TCA, and RCA consume signature generation times of 80.66 ms, 50.27 ms, 5.035 ms, 5.035 ms, and 5.079 ms, respectively. Therefore, the proposed methods significantly reduce the signature verification time by 10–16 times, as compared to the two previous methods.

In summary, the reduction ratio in communication and computation overhead that the proposed methods provide, tends to rapidly increase for a large-scale network with a large number of neighboring zones. In our future work, we intend to improve the proposed authentication methods by integrating them into different 5G applications, as recommended by the authors of [38]. We also intend to implement the proposed authentication methods using hardware devices and compare the performance with the results of our previous decentralized hash-chain-based protocol [39].

## 7. Conclusions

In this paper, we presented three authentication methods for multi-zone communications, based on the bilinear pairing cryptography and short signatures. The 5G-V2X standards support the installation of many base stations at short distances, which can be utilized to provide a dynamic key generation and multi-hop authentication for vehicles. In this paper, we divide the network into N zones, each zone covered by n BSs. Each vehicle communicates securely using different keys per zone, which enhances the security level and supports updated keys through different zones. In the proposed TCA method, the signature generation and verification depend on the transmitter zone parameters. In the proposed SCA method, the transmitter generates a concatenated signature that can be verified individually by all receivers, using their corresponding zone parameters. Transmitters and receivers in the RCA method aggregate the security parameters of the communicated neighboring zones to generate and verify signatures. The proposed three authentication methods support message signing and verification at a low cost, using short signatures over bilinear pairing curves. We compared the communication and computation cost of the proposed authentication methods and six previous methods for single-zone and multi-zone scenarios. The proposed methods significantly reduce the signature generation time by 16 times–80 times, as compared to the compared previous methods. Additionally, the proposed methods significantly reduce the signature verification time by 10 times–16 times, as compared to the two previous methods. The three proposed authentication methods achieved substantial speed-up in the signature generation time and verification time, using a short bilinear pairing signature.

## Figures and Tables

**Table 1:** Comparison of the previous certificateless bilinear pairing authentication methods.

Message Authentication Done by Signing Message Using Individual Secret Keys and Verification is Done Using the Bilinear Pairing Function. IDENTITY Authentication Is Satisfied Using Pseudo-Identities			
Certificateless Bilinear Pairing Cryptography Is Used in All Compared Methods [19,20,21,22,23,24] for Single Group Communication			
Security Method	Advantages	Disadvantages	Communication Type
Horng et al. [19]	The user’s private keys are not stored at the Key Generator Center (KGC) Support signatures aggregation Self-generation of private keys	The proposed security model cannot resist the passive malicious Key Generator Center (KGC) attacks	Support Vehicle-to-Infrastructure (V2I) communication
Li et al. [20]	They proposed a proof and analysis of the Horng et al. [19] method They prove that [19] does not resist malicious-but-passive KGC attacks They support signatures aggregation at Road side Units (RSUs)	Their method introduce additional communication cost The dependence on a fully trusted third party	Support Vehicle -to-Infrastructure (V2I) communication
Malhi et al. [21]	They proposed a new efficient certificateless aggregate signature protocol They proved the security level using the random oracle model Computationally more efficient due to its constant pairing operations	Their method introduce additional communication cost Aggregation of signatures done at vehicles by aggregate the messages related to the same Road Side Units Vehicles work as aggregator generator	Support ad hoc communication
Lin et al. [22]	Single manager issues the secret keys for vehicles Resist the KGC attacks Support signature aggregation Propose a secure protocol based on group signature and identity (ID)-based signature techniques	Introduce high computation time due to excessive use of bilinear pairing operations Unfortunately this method is vulnerable to the impersonation attack	Support Vehicle-to-Vehicle (V2V) communication Support V2-I communication
Bayat et al. [23]	They proposed a new Conditional Privacy-Preserving Authentication (CPPA) method based on the bilinear pairing cryptography They improved the identity-based authentication in V2X. They analyze a recent authentication scheme for VANETs introduced by Lee et al.	However, their method cannot prevent the message modification attacks in which an attacker can repeat the transmission of old messages after modifying its content.	Support V2V and V2I communications
Boneh et al. [24]	They proposed a group signature-based on bilinear pairing.	Introduce high communication cost Suffers from high verification time at receivers due to high number of bilinear pairing operations	Support V2V communication Support V2-I communication

**Table 2:** The system notations and abbreviations.

Notations	Descriptions
BSs	Base Stations
CA	Certificate Authority
ECC	Elliptic Curve Cryptography
$Lpid_{i}$	a list of pseudo identities $\left(pid_{i}{}_{1},\;pid_{i}{}_{2},\;pid_{i}{}_{3}\ldots ,pid_{i}{}_{n}\;\right)$
$Lsk_{i}$	list of secret keys$\;\left(sk_{i}{}_{1},\;sk_{i}{}_{2},\;sk_{i}{}_{3},\ldots ,sk_{i}{}_{n}\;\right)$
$Lpk_{i}$	a list of the corresponding public keys $\left(pk_{i}{}_{1},pk_{i}{}_{2},pk_{i}{}_{3},\ldots ,pk_{i}{}_{n}\right)$
$L_{ZID}$	Zone of ID list
$\;sk_{i}{}_{ZID}$	A random integer number represents a secret key of vehicle $v_{i}\;$in each zone
$F_{p}$	Finite field of elements in the range {1 and p−1}.
$G_{1},G_{2}$	two cyclic additive groups of prime order p based on the elliptic curve E over the finite field$\;F_{p}$ where $G_{1}\times G_{2}$ *→* $G_{T}$
$G_{T}$	Acyclic multiplicative group containing the bilinear pairing result of the two groups $G_{1},G_{2}$.
e	The bilinear pairing function that maps elements from group $G_{1}$ and group $G_{2}$ to group $G_{T}$
$\;g_{2}{}_{ZID}$	Represents the generator point of the group$\;G_{2}$ for each zone
$pk_{i}{}_{ZID}$	An ECC point represents the public key of vehicle $v_{i}\;$in each zone:$\;sk_{i}{}_{ZID}.\;g_{2}{}_{ZID}$
H	A cryptographic hash function that maps a message to a point in the group ${\;G}_{1}$
$pid_{i}$	Represents the pseudo-identity of $v_{i}$ to hide it’s real identity and allow vehicle to communicate anonymously
$m_{i}$	Represents message payload transmitted from vehicle $v_{i}$
$T_{s}$	Is a timestamp to ensure message freshness
n	The number of base stations
N	The number of zones
H(m)	Hashed message to a point over the elliptic curve group $G_{1}$
$\sigma_{i}$	Generate a signatureover message m using the secret key of each vehicle
||	Represents the concatenation operation of two elements
$g_{2}{}_{aggr}$	Represents the aggregation of zones generators ($\;g_{2}{}_{ZID})$ to generate a new value$\;\in G_{2}:\;{g_{2}}_{1}+\;{g_{2}}_{2}+\ldots +\;{g_{2}}_{N}$
$sk_{aggr}$	Represents the aggregation of vehicle $v_{i}$ secret keys for different zone destinations $sk_{1}+sk_{2}+\ldots +sk_{N}$ $\in \;F_{p}$
$pk_{aggr}$	Represents the aggregated public key of vehicle$\;v_{i}:sk_{aggr}\;.g_{2}{}_{aggr},$ where $pk_{aggr}$ $\in G_{2}\;$
$\sigma_{aggr}$	The aggregated signature over message m using the aggregated secret key $sk_{aggr}:\;sk_{aggr}\;.H\left(m\right)$, where $\sigma_{aggr}\in G_{1}$
$\sigma_{C}$	The concatenated signatures that consists of N signatures generated by vehicle $\;v_{i}\;$for different zones destinations: $\left(\sigma_{1}||\;\sigma_{2},\ldots ||\sigma_{N}\right)$
$pk_{C}$	The concatenated public keys that consists of N public keys of vehicle $\;v_{i}$ for different zones destinations: $\left(pk_{1}||\;pk_{2},\ldots ||pk_{N}\right)$

**Table 3:** The BN256 bilinear pairing curve parameters.

Pairing Curve equation	E: $y^{2}=x^{3}+b\;$
Size of elements in the finite field $F_{p}$	|$F_{p}$| = 32 bytes
b	Integer number over$\;F_{p}$ where b = 2
Size of elements in the elliptic curve group $G_{1}$	|$G_{1}$| = 32 bytes
Size of elements in the elliptic curve group $G_{2}$	|$G_{2}$| = 2 |$G_{1}$| = 64 bytes
Size of elements in the result mapping group $G_{T}$	|$G_{T}$| = 12 |$G_{1}$| = 384 bytes

**Table 4:** Average execution time of the BN254 pairing functions used in our simulation.

Operation	Definition	Time in (ms)
Bilinear pairing	The time needed to perform one bilinear pairing of elements from group $G_{1}$ and group $G_{2}$ to group $G_{T}$	TP = 2.446
Addition in $G_{1}$	The time of addition of two points inside group $G_{1}$	TAG1 = 0.007
Multiplication in $G_{1}$	Scalar multiplication of a point inside group $G_{1}$and a random integer	TMG1 = 0.479
Addition in $G_{2}$	The time of addition of two points inside group $G_{2}$	TAG2 = 0.013
Multiplication in $G_{2}$	Scalar multiplication of a point inside group $G_{2}$ and a random integer	TMG2 = 0.989
HashAndMap to $\;G_{1}$ or $G_{2}$	The time of hashing message using sha-256 then map the hashed result to a point in the group $G_{1}$ or group $G_{2}$	TM2P = 0.135
Hashing Operation	the time defined for one hash function operation using SHA-256 algorithm	TH = 0.006
Addition in $F_{p}$	The time of addition of two points over the finite filed $F_{p}$	TAFp = 0.001

**Table 5:** A comparison of signature generation time and verification time for the proposed authentication methods and the previous methods in single-zone and multi-zone scenarios.

Security Methods	Signature Generation Time Per Message for Single Zone Case (ms)	Signature Verification Time Per Message for Single Zone Case (ms)	Signature Generation Time Per Message for n Receivers in n Multi-Zones (ms)	Signature Verification Time Per Message for Each Receiver in n Multi-Zones (ms)
(1) Horng et al. [19]	2TMG1 + 2TAG1 = 0.9594	3TP + TMG1 + TM2P = 7.8305	0.9594n	3 nTP + nTMG1 + nTM2P = 952.7n
(2) Li et al. [20]	TAG1 + 2TMG1 + TM2P = 1.1	3TP + TMG1 + 2TM2P + TAG1 = 8.094	1.1n	3nTP + nTMG1 + 2nTM2P + nTAG1 = 8.094n
(3) Malhi et al. [21]	4TMG1 + 2TAG1 = 1.93	3TP + 3TMG1 + TAG1 = 8.782	1.93n	3nTP + 3nTMG1 + nTAG1 = 8.778n
(4) Lin et al. [22]	3TP + TM2P = 7.473	5TP + 8TMG1 = 16.132	7.473n	5nTP + 8nTMG1 = 16.132n
(5) Bayat et al. [23]	5TMG1 + TAG1 + TH + TM2P = 2.549	3TP + TMG1 + TH + TM2P = 7.8365	2.549n	3n TP + nTMG1 + nTM2P = 7.952n
(6) Boneh et al. [24]	3TP + 5TMG1 = 9.7275	4TP + 2TM2P = 10.054	9.7275n	4nTP + 2nTM2P = 10.054n
The proposed TCA	TM2P + TMG1 = 0.614	2TP + TM2P = 4.9 + 0.135 = 5.035	0.614	2TP = 5.035
The proposed SCA	TM2P + TMG1 = 0.614	2TP + TM2P = 4.9 + 0.135 = 5.035	0.614n	2TP = 5.035
The proposed RCA	(n−1 )TAFp+(n−1 )TAG2+TM2P + TMG1 = 0.600 + 0.014n	(n−1) TAG2+TM2P+2TP=0.013 (n−1)+0.135+4.9=5.014+0.013n	0.600 + 0.014n	5.014 + 0.013n
